# The autosomal *Gsdf* gene plays a role in male gonad development in Chinese tongue sole (*Cynoglossus semilaevis*)

**DOI:** 10.1038/s41598-018-35553-7

**Published:** 2018-12-07

**Authors:** Ying Zhu, Liang Meng, Wenteng Xu, Zhongkai Cui, Nianwei Zhang, Hua Guo, Na Wang, Changwei Shao, Songlin Chen

**Affiliations:** 1 Key Lab of Sustainable Development of Marine Fisheries, Ministry of Agriculture, Yellow Sea Fisheries Research Institute, Chinese Academy of Fishery Sciences, Qingdao, 266071 China; 2 Laboratory for Marine Fisheries Science and Food Production Processes, Qingdao National Laboratory for Marine Science and Technology, Qingdao, 266237 China

## Abstract

*Gsdf* is a key gene for testicular differentiation in teleost. However, little is known about the function of *Gsdf* in Chinese tongue sole (*Cynoglossus semilaevis*). In this study, we obtained the full-length *Gsdf* gene (*CS*-*Gsdf*), and functional characterization revealed its potential participation during germ cell differentiation in testes. *CS*-*Gsdf* transcription was predominantly detected in gonads, while the levels in testes were significantly higher than those in ovaries. During the different developmental stages in male gonads, the mRNA level was significantly upregulated at 86 dph, and a peak appeared at 120 dph; then, the level decreased at 1 and 2 yph. *In situ* hybridization revealed that *CS-Gsdf* mRNA was mainly localized in the Sertoli cells, spermatogonia, and spermatids in mature testes. After *CS-Gsdf* knockdown in the male testes cell line by RNA interference, a series of sex-related genes was influenced, including several sex differentiation genes, *CS-Wnt4a*, *CS-Cyp19a1a* and *CS-Star*. Based on these data, we speculated that *CS-Gsdf* may play a positive role in germ differentiation and proliferation via influencing genes related to sex differentiation.

## Introduction

In teleost, several genes that belong to TGF-β signal components have been identified to have sex-determination functions, including *Amhy*, *Amhr2*, *Gsdf*^*Y*^ and *Gdf6*^*Y*^^1–5^. Studies on these genes have focused on *Gsdf* (gonadal soma-derived factor), including its specific expression in teleosts, such as *Danio rerio*, *Takifugu rubripes* and *Oryzias latipes*^2,6–8^. In general, *Gsdf* performs vital functions in male germ cell proliferation and testicular differentiation^6,8,9^, and as a teleost-specific gene, it is predominantly expressed in Sertoli cells and surrounding cells in mature gonads of *Oryzias luzonensis* and *Oncorhynchus mykiss*^3,10^. In addition, in *O. luzonensis*, a Y-chromosome localized *Gsdf* was reported to be the male-determining gene^3^, and its deletion could cause feminization^11,12^. *Gsdf* has been shown to be an excellent candidate for understanding the sex-determination events in *Anoplopoma fimbria*^13^.

Despite the divergent role, accumulated data now support that autosomal *Gsdf* functions as a male sex initiator and initiate testicular differentiation, which has been proven to be an early marker in the gonads of males in *Oryzias latipes* and *Oreochromis niloticus*^6,11,14,15^. As *Dmy*-independent sex-determining gene during sex-chromosome evolution^16–18^, *Gsdf* could play an important role in sex differentiation by interacting with *Dmy* or *Dmrt1*, and affecting oestrogen production^19,20^. Additional features of the *Gsdf* gene included the regulation of primordial germ cell proliferation and meiotic germ cell proliferation or differentiation^6,10^.

Chinese tongue sole (*Cynoglossus semilaevis*) is an economically important marine flatfish that is widely cultured in China. This species exhibits sexual growth dimorphism and females grow 2–4 times faster than males^21^; thus, increasing the proportion of females would increase the culturing productivity. However, many limiting factors result in low female ratios in aquaculture; for instance, genetic females (ZW) could sex-reverse to phenotypic males under some conditions^22,23^. In Chinese tongue sole, several genes have been reported to be involved in male sex determination and differentiation, including *Dmrt1*, *Tesk1*, *Piwil2* and *Neurl3*^24–27^. Among these genes, *Dmrt1* was previously demonstrated to be the male-determining gene^22,24^, while the others were found to be involved in spermatogenesis. More and more reports focused on the relationship between environmental factors and sex differentiation/gonad development^28,29^. Although, in our lab, epigenomics data in Shao *et al*. revealed that *Gsdf* is an important sex-related gene, its expression is mediated by DNA methylation in *C. semilaevis*^30^, while little is known about the features and functions of the *Gsdf* gene.

To investigate the role of *Gsdf* in fish with the ZW sex-determining system, in this study, we first cloned the full cDNA sequence of *CS-Gsdf*. We then analysed the *Gsdf* expression patterns in gonads at different developmental stages by qRT-PCR and its special distribution in gonads via *in situ* hybridization (ISH). Furthermore, RNA interference (RNAi) of *CS-Gsdf* was performed in the testicular cell line, and a series of sex-related genes were analysed.

## Results

### Analysis of *CS-Gsdf* cDNA sequence

**Figure 1:**
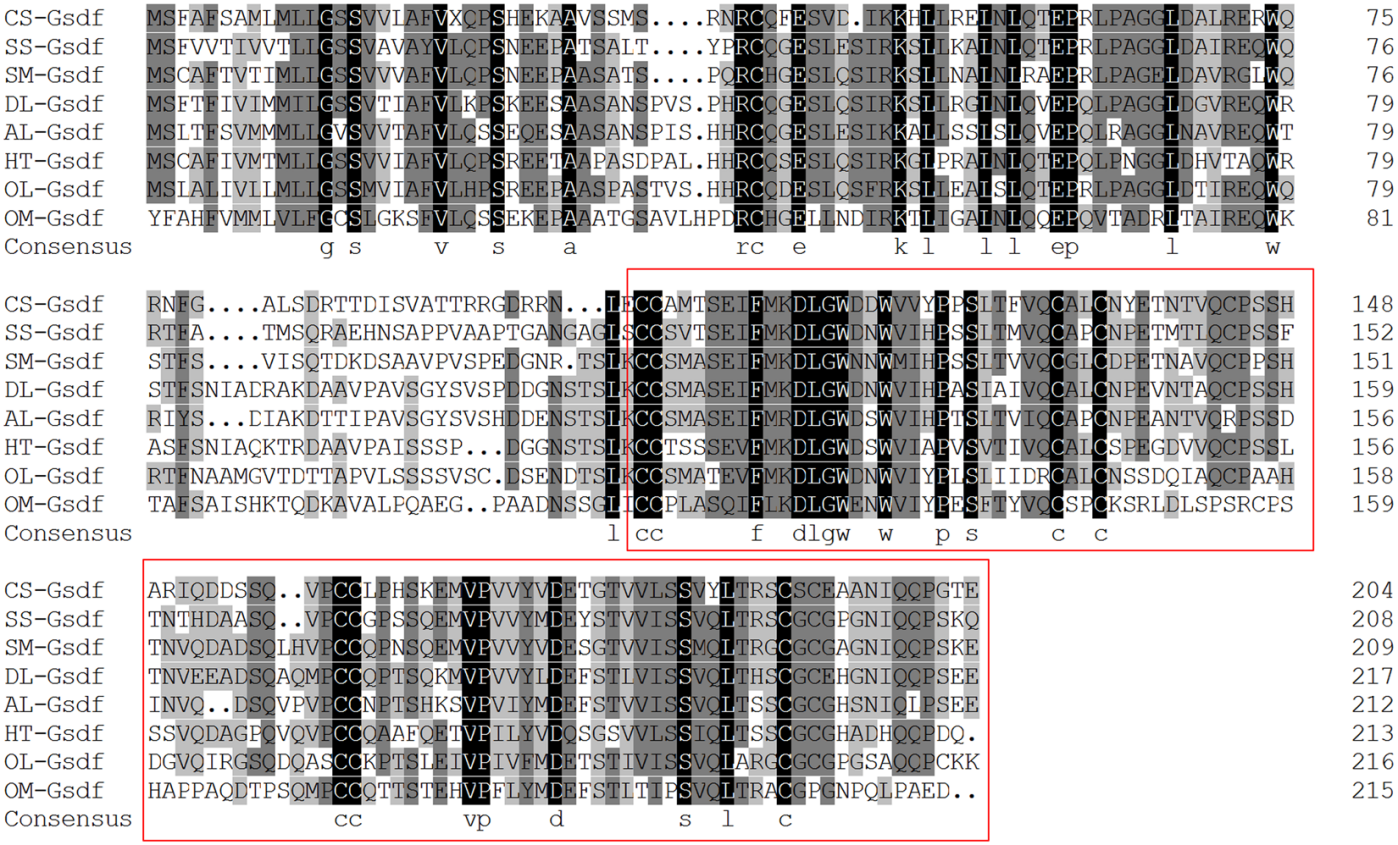
Multiple alignment of *C*. *semilaevis* Gsdf protein sequences with other teleosts. Sequences are aligned using ClustalX and DNAMAN. The presumed TGF-β domain region is indicated by the red box. The abbreviations of protein names used in this section are as follows: CS-Gsdf: *Cynoglossus semilaevis* Gsdf; SS-Gsdf: *Solea senegalensis* Gsdf; SM-Gsdf: *Scophthalmus maximus* Gsdf; DL-Gsdf: *Dicentrarchus labrax* Gsdf; AL-Gsdf: *Acanthopagrus latus* Gsdf; HT-Gsdf: *Halichoeres trimaculatus* Gsdf; OL-Gsdf: *Oryzias latipes* Gsdf; OM-Gsdf: *Oncorhynchus mykiss* Gsdf.

The complete cDNA sequence is 1,244 bp in length, containing a 139 bp 5′ untranslated region (UTR), a 615 bp open reading frame (ORF) and a 470 bp 3′ UTR. The ORF encoded a putative protein with 204 amino acids (GenBank accession number: MG891889) (Figs. 1, S1 and S2). The putative protein has a predicted molecular weight of 22.75 kDa and a theoretical isoelectric point of 5.66.

### Expression patterns of *CS-Gsdf* in Chinese tongue sole

**Figure 2:**
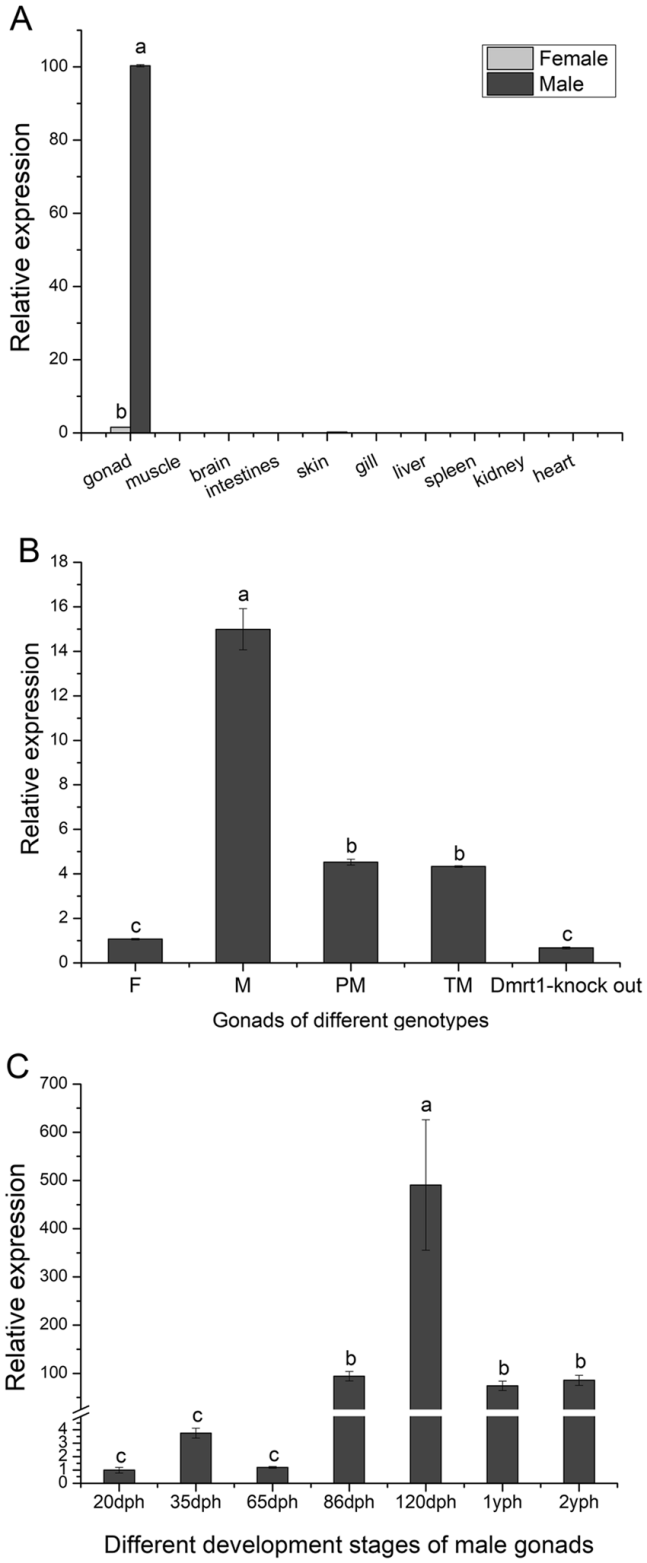
Expression analysis of *CS-Gsdf* in *C. semilaevis* evaluated by qRT-PCR. (**A**) *CS*-*Gsdf* transcription in various tissues of *C. semilaevis*. (**B**) *CS-Gsdf* transcription in the gonads of different sexual genotypes. (**C**) *CS-Gsdf* transcription in the male gonads at different developmental stages. F: female, M: male, PM: pseudo-male, TM: triploid male. *Dmrt1*-knockout: *Dmrt1*-knockout fish. The transcription levels were normalized using the *β-actin* levels. The bars represent the triplicate mean ± SEM values from three separate individuals (n = 3). The different letters on bars denote statistical significance (*p* < *0.05*).

To determine the tissue distribution of *CS-Gsdf* in Chinese tongue sole, we analysed the expression levels in 10 different tissues of 1-year post-hatching female and male tongue sole by qRT-PCR. *CS-Gsdf* was expressed in only the gonads, and the expression level was much higher in the testis than that in the ovary (Fig. 2A).

Using qRT-PCR, we also examined the expression levels in the gonads of male, female, pseudo-male, triploid male (infertile) and *Dmrt1*-knockout fish. The highest *CS-Gsdf* mRNA transcript level was detected in the gonads of males, while the mRNA expression levels of *CS-Gsdf* were low in the pseudo-male and triploid male. The lowest expression levels were observed in the gonads of the female and *Dmrt1*-knockout fish (Fig. 2B).

To study the expression of *CS-Gsdf* during the differentiation and development of male gonads, we measured the expression levels in testes at different developmental stages. As shown in Fig. 2C, the *CS-Gsdf* transcripts were detected at 20 days post-hatching (dph) and continued to be expressed at very low levels before sharply increasing at 86 dph. At 120 dph, the expression reached its peak in the testis and then declined at 1-year post-hatching (yph) and 2 yph.

### Cyto-localization of *CS-Gsdf* mRNA in the gonads

**Figure 3:**
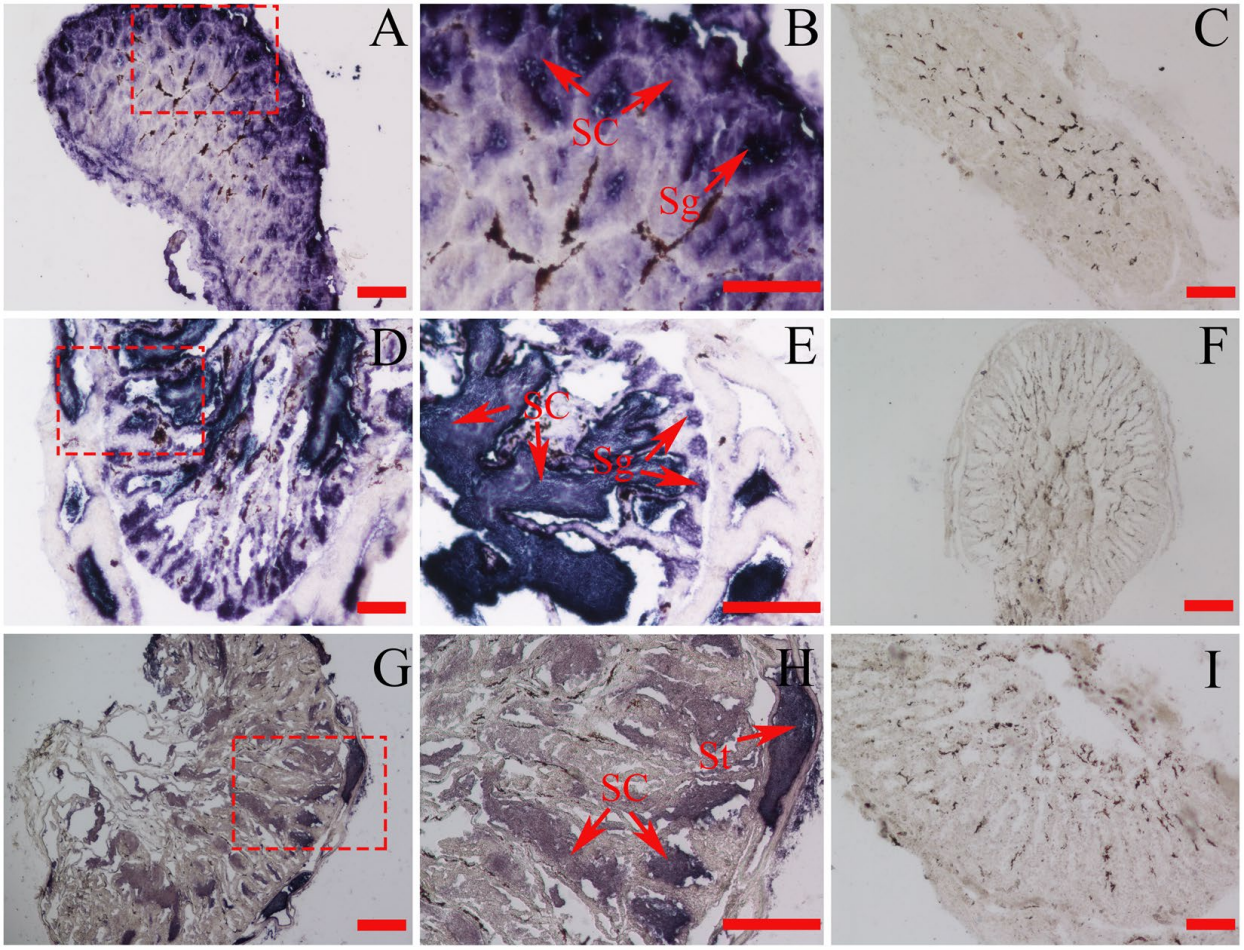
*In situ* localization of *CS-Gsdf* mRNA in the gonads of *C. semilaevis*. (**A**) Testis of a male at 120 dph. (**B**) Magnification of the red framed area in A. (**C**) Testis of a male at 120 dph with sense probes as a control. (**D**) Testis of a male at 1 yph. (**E**) Magnification of the red framed area in D. (**F**) Testis of a male at 1 yph with sense probes as a control. (**G**) Testis of a male at 2 yph. (**H**) Magnification of the red framed area in G. (**I**) Testis of a male at 2 yph with sense probes as a control. Sg: spermatogonia, SC: Sertoli cells, St: spermatids, Bars = 100 μm.

The ISH results demonstrated that *CS-Gsdf* was mainly expressed in Sertoli cells, spermatogonia and spermatids with intensive hybridization signals at 120 dph and 1 yph (Fig. 3A, B, D and E). In contrast, faint signals were detected in the spermatids of the 2 yph testes (Fig. 3G and H). As a negative control, no positive signal was detected in the hybridization sections with sense probes (Fig. 3C, F and I).

### RNAi-mediated *CS-Gsdf* knockdown and its impact on the mRNA expression of sex-related genes

**Figure 4:**
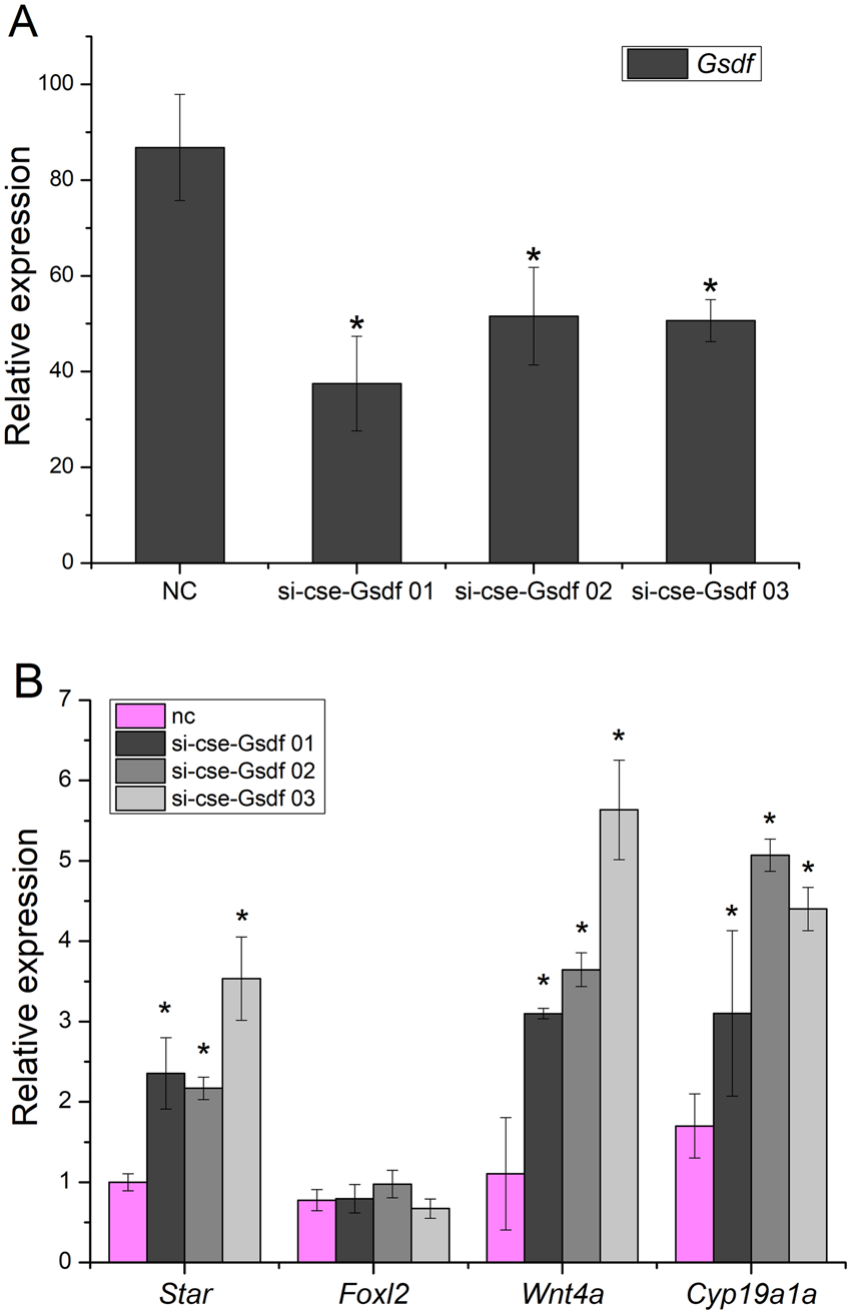
The analysis of *Gsdf*, *Star*, *Foxl2*, *Wnt4a* and *Cyp19a1a* expression in cultured testis cells after RNAi. (**A**) Expression of *CS-Gsdf* after the transfection of siRNA at 48 h. (**B**) Expression levels of *CS-Star*, *CS-Foxl2*, *CS-Wnt4a* and *CS-Cyp19a1a* were determined by qRT-PCR after the transfection of the siRNA for 48 h. NC, si-001, si-002 and si-003 indicate the testis cells transfected with the siRNA of the negative control (NC) interfered with the 001 sites, 002 sites and 003 sites, respectively. Asterisks indicate significant differences (*p* < 0.05) between the treated group and the control.

The RNAi-mediated knockdown *in vitro* was conducted for *CS-Gsdf* in a Chinese tongue sole testicular cell line (CSGC). To determine the silencing effects of RNAi, *CS-Gsdf* expression in CSGC was detected by qRT-PCR 48 h after siRNA transfection. The results revealed that the silencing efficiencies of *CS-Gsdf* were approximately 79.1%, 70.9% and 71.3% in the si-cse-*Gsdf* 01, 02 and 03 treatments, respectively (Fig. 4A).

To evaluate the effects of *CS-Gsdf* RNAi-mediated knockdown on the expression of sex-related genes, *Foxl2*, *Star*, *Wnt4a*, and *Cyp19a1a* were measured. As shown in Fig. 4B, compared with the control, the expression levels of *CS*-*Gsdf*, *CS*-*Foxl2*, *CS*-*Star*, *CS*-*Wnt4a*, and *CS*-*Cyp19a1a* were detected by qRT-PCR.

## Discussion

*Gsdf* belongs to the transforming growth factor-beta (TGF-beta) family, which is important for the regulation of cell growth, differentiation, and migration^31,32^. Moreover, recent reports found that members of the TGF-beta family may have a function in the sex-determining process^33^. To determine the role of *CS*-*Gsdf* in tongue sole, we initiated this study.

In this work, we cloned and characterized *CS-Gsdf* cDNA. The predicted protein contained a highly conserved TGF-beta region, which is characterized by 7 conservative cysteine residues called the conserved cysteine knot motif (Fig. 1), which is not present in trout lacking 6th cysteine residues^6^. Different from other TGF-β family members, Gsdf lacks glycine residues in the conserved motif^34^.

The expression analysis revealed that *CS-Gsdf* was expressed in only gonads, and the expression level was much higher in testes than that in ovaries. The threshold of *CS-Gsdf* mRNA transcription was observed at 20 dph, which is consistent with the previous reports of its expression in early teleost stages, such as that observed at 5 days post-fertilization (dpf) in *O. latipes*, 16 dpf in *D. rerio*^6,35^ and 30 dpf in *O. mykiss*^10^, which are all prior to testicular differentiation^6,17^. As histological differentiation occurred at 50–65 dph in *C. semilaevis*, and 70–90 dph were selected to represent appearance of oogonium/spermatogonia, whereas cellular differentiation occurred at 120–150 dph, including oocyte/spermatocyte and so on^21,36,37^. *CS-Gsdf* exhibited high expression at 86 dph and peak expression at 120 dph in testes. However, the expression subsequently declined in mature testes which nearly completed cellular differentiation and seminiferous tubule formation^20,22,38,39^. During germ cells divide rapidly, and Sertoli cells actively regulate the surrounding congregated spermatogonia^22,23,40^. Thus, the relatively strong signals in Sertoli cells suggested steroidogenesis and spermatogenesis functions^41–44^. Furthermore, the lowest *CS-Gsdf* expression levels were found in *Dmrt1*-deficient tongue sole (Fig. 2B), and few spermatogonia were observed in this sample. Similar findings in *Oreochromis niloticus* have been reported^45^. It has been widely reported that *Gsdf* exists in Sertoli cells in *O. mykiss*^10^, *D. rerio*^35^, *O. luzonensis*^3^, *O. sakaizumii*^20^, *Monopterus albus*^38^, and *Paralichthys olivaceus*^39^. Given these findings, we speculated that *CS-Gsdf* probably plays an important role in germ cell differentiation and proliferation.

After the *in vitro* knockdown of *CS-Gsdf* in cultured male cells (Fig. 4A), qRT-PCR showed the mRNA levels of the sex-related genes *CS-Star*, *CS*-*Cyp19a1a*, and *CS*-*Wnt4a*, which all increased after *CS*-*Gsdf* knockdown except *CS*-*Foxl2*. While, when *CS*-*Dmrt1* is knocked out, both *CS*-*Cyp19a1a* and *CS*-*Foxl2* are significantly upregulated in tongue sole^24^. *Cyp19a1a* is both the sex-determining gene and the regulator of steroid hormone synthesis in teleosts, and this gene plays a role in gonadal differentiation. Meanwhile, *Star* is a rate-limiting step that mediates steroid hormone synthesis^46–52^. Therefore, we speculated that *CS*-*Gsdf* was involved in sex differentiation through mediating genes related to gonadal hormones. Similar results appeared in two types of medaka (*O. latipes* and *O. sakaizumii*), which proved *Gsdf* affected oestrogen production during sex differentiation^19,20^. Simultaneously, *Wnt4a* is a key gene in the *Wnt4/β-catenin1* pathway that regulates gonad development/differentiation^53,54^. *CS*-*wnt4a* was also upregulated after RNAi, although the mechanisms remain elusive. Interestingly, *CS-Gsdf* obviously declined in *Dmrt1*-deficient gonads, which might be positively regulated by sex-determination genes. In conclusion, *CS*-*Gsdf* transcription could affect gene expression related to gonadal hormone genes but exert a negative effect on female *Foxl2* gene.

## Conclusion

In summary, we cloned and characterized *CS*-*Gsdf* from Chinese tongue sole. *CS-Gsdf* was specifically expressed in gonads, with much higher expression levels in testes than those in ovaries. In testes, the threshold of *CS-Gsdf* transcription was detected at 20 dph, increased at 86 dph, and peaked at 120 dph, Its mRNA was mainly localized in Sertoli cell and spermatogonia. After *in vitro* RNAi in the testicular cell line, several sex-related genes were affected. Based on these data, we proposed that *CS*-*Gsdf* participates in germ cells differentiation and proliferation of testes, while further studies are needed to elucidate its detailed role.

## Materials and Methods

### Ethics statement

The handling of experimental fish was approved by the Animal Care and Use Committee of the Chinese Academy of Fishery Sciences, and all protocols were performed in accordance with the guidelines of the Animal Care and Use Committee. To minimize fish suffering, tissues were collected under MS222 anaesthesia.

### Experimental fish preparation and sample collection

The Chinese tongue sole used in this study were purchased from the Haiyang High-Tech Experimental Base (Haiyang, Shandong Province, China). Temperature treatments were performed to induce pseudo-males, as previously described^55^. Ten individuals of each scope (including males, females and pseudo-males) participated in this work. The brain, heart, intestine, gill, kidney, liver, muscle, skin, spleen, and gonads were collected from 1-year-old fish, immediately subjected to liquid nitrogen and then stored at −80 °C until RNA extraction. The gonads at different developmental stages (at 20, 35, 65, 86, 120 dph, 1 and 2 yph) were picked from one side and frozen in liquid nitrogen until RNA extraction. The contralateral gonads were also picked and divided into two sections: one was placed in 4% paraformaldehyde (PFA) for *in situ* hybridization (ISH), and the other was simultaneously placed in Bouin’s fixative for histological analysis of phenotypic sex. The tail fins of all experimental fish were collected and preserved in 100% ethanol for DNA extraction and subsequent genetic sex determination.

### DNA, RNA extraction and cDNA synthesis

The process used to extract genomic DNA followed the standard phenol-chloroform extraction method^56^, which was then used as a template for subsequent analysis after quantification.

Total RNA was extracted using TRIzol reagent (Invitrogen, Carlsbad, CA, USA) and then quantified by NanoVue Plus (BiochRom LTD, Cambridge, England). The first-strand cDNA was synthesized using a PrimeScript RT reagent Kit with gDNA Eraser (TaKaRa, City, Country). A total of 800 ng of total RNA from each sample was reverse transcribed into first-strand cDNA and used as the template for qRT-PCR.

### Identification of phenotypic and genetic sex

To verify the phenotypic sex of all experimental individuals, gonadal histology was carried out as previously described^57^. The genetic sex was determined by following the methods of Liu *et al*.^58^. The sex-specific simple sequence repeat (SSR) markers scaffold68-2F and scaffold68-2R (Table S1) were designed for PCR amplification as previously described^58^.

### Isolation of *CS-Gsdf* full-length cDNA

To obtain the full-length cDNA of *CS*-*Gsdf*, rapid amplification of cDNA ends (RACE) was performed using the SMART RACE cDNA Amplification Kit (Clontech Inc., Mountain View, CA, USA). RACE-ready first-strand cDNA was synthesized from total RNA according to the manufacturer’s instructions, and the gene-specific primers for outer and nest amplification were designed (Table S1). The outer amplification was performed using touchdown PCR procedures as described by Meng^25^. PCR products were electrophoresed on a 1.0% agarose gel, and the amplified fragments of expected size were depurated with a Zymo clean Gel DNA Recovery Kit (ZYMO Research, Orange, CA, USA). Purified products were cloned into a pMD18-T vector (TaKaRa, Dalian, China) and sequenced.

### Analysis of qPCR

QRT-PCR primers (Table S1) were designed based on the *CS-Gsdf* cDNA sequence, and their specificity was verified by a single distinct peak obtained in a melting curve analysis. QRT-PCR was conducted using a 7500 ABI real-time PCR system (Applied Biosystems) with SYBR Green Master Mix (TaKaRa). β-actin was used as the internal control^59^. Three randomly selected individuals were subjected to qRT-PCR, and the experiment was performed in triplicate for each sample.

The relative mRNA expression of target genes was calculated by the 2^−ΔΔCt^ method. All data were tested using one-way ANOVA followed by Duncan multiple comparison tests using SPSS 18.0 (IBM, New York, NY, USA). Significance was accepted only when p < 0.05. All assays in the qRT-PCR complied with the MIQE guidelines^60^.

### *In situ* hybridization

After dehydration in ethanol, the stored gonad samples were fixed in paraffin wax and sheared as 5 µm sections. A pair of primers (Table S1) for RNA probe synthesis was designed according to the *CS*-*Gsdf* ORF sequence. The PCR product was cloned into a pBluescriptSKII plasmid and then linearized with *Pst*I and *Sal*I (TaKaRa). Probes were labelled using DIG RNA Labeling Mix (Roche, Mannheim, Germany). The ISH was performed following a previously described method^61^ using samples from three different individuals. Images were captured with a Nikon E80i microscope (Nikon, Tokyo, Japan) and then analysed.

### *In vitro* RNAi of Gsdf

The three *Gsdf*-specific small interfering RNAs (si-cse-*Gsdf* 01, 02 and 03) were designed and synthesized by RayBiotech C. Ltd. In addition, a nonspecific siRNA negative was used as a control (NC) during the experiment (Guangzhou, Guangdong province, China). The testicular (CSGC) cell line, which was previously created in our laboratory, was employed for RNAi silencing. The CSGC cells were recovered as described by Zhang *et al.*^62^, and then transferred to six-well plates. After cultivating at 24 °C for 12 h, the cells completely attached to the plates, and then the labelled siRNAs were transfected into the cells using Lipofectamine 2000 reagent (Invitrogen) according to the manufacturer’s instructions. The calculated average transfection efficiency was approximately 80%. Both the treated groups (using si-cse-*Gsdf* 01, 02 and 03) and the control group (using NC siRNA) were transfected at a concentration of 30 nM. The collected cells were cultivated at 24 °C for 48 h. The total RNA was extracted from the cells, and cDNA was synthesized as described above. The relative expression levels of the genes related to sex differentiation, such as *Star*, *Cyp19a1a*, *Foxl2* and *Wnt4a*, were evaluated by qRT-PCR, and all experiments were performed in triplicate.

## Electronic supplementary material

Supplementary Information
