# Supplementary material for: The autosomal Gsdf gene plays a role in male gonad development in Chinese tongue sole (Cynoglossus semilaevis)
Source: Sci Rep. 2018 Dec 7;8:17716. doi: 10.1038/s41598-018-35553-7 (PMC6286346; doi:10.1038/s41598-018-35553-7)
Supplement: Supplementary file 1 — Supplementary Information [file 41598_2018_35553_MOESM1_ESM.pdf]

The autosomal *Gsdf* gene plays a role in male gonad development in Chinese tongue sole (*Cynoglossus semilaevis*):

Supplementary Information

Ying Zhu[1,2 +], Liang Meng[1,2 +], Wenteng Xu[1,2], Zhongkai Cui[1,2], Nianwei Zhang[1,2], Hua Guo[1,2], Na Wang[1,2], Changwei Shao[1,2], Songlin Chen[1,2*]

1 Key Lab of Sustainable Development of Marine Fisheries, Ministry of Agriculture; Yellow Sea Fisheries Research Institute, Chinese Academy of Fishery Sciences, Qingdao 266071, China

2 Laboratory for Marine Fisheries Science and Food Production Processes, Qingdao National Laboratory for Marine Science and Technology, Qingdao 266237, China

+The first two authors contributed equally to this paper.

*Corresponding author: S.C. (chensl@ysfri.ac.cn)

```
                                                                                                          acatggggaatacggcgta      19
gagcatcctggtctctacacgtccatctgcaacctccaacctgacacgcaccaccgagccgctgcagaattcttctgatttgaaagcgtctcatctggcagacccttcaccttggccacc     139
ATGTCATTTGCATTCAGTGCCATGCTAATGCTCCTGGGTTCTTCGGTTGTGCTTGCGTTTGTCTTGCAGCCGTCCCATGAAAAAGCTGCAGTCTCTTCTATGTCTCGCAACAGGTGCCAG     259
 M  S  F  A  F  S  A  M  L  M  L  L  G  S  S  V  V  L  A  F  V  L  Q  P  S  H  E  K  A  A  V  S  S  M  S  R  N  R  C  Q   40
TTTGAGTCAGTGGACATCAAGAAGCATCTCCTCAGAGAACTGAACCTCCAGACTGAGCCGCGTCTTCCTGCTGGTGGTCTGGATGCTCTCAGAGAGAGATGGCAGAGGAACTTTGGCGCC     379
 F  E  S  V  D  I  K  K  H  L  L  R  E  L  N  L  Q  T  E  P  R  L  P  A  G  G  L  D  A  L  R  E  R  W  Q  R  N  F  G  A   80
TTGTCTGACAGAACCACGGACATTTCAGTTGCCACAACCAGGCGTGGAGACAGAAGGAACCTGGAGTGTTGCGCCATGACGTCTGAGATTTTCATGAAAGATCTGGGCTGGGACGACTGG     499
 L  S  D  R  T  T  D  I  S  V  A  T  T  R  R  G  D  R  R  N  L  E  C  C  A  M  T  S  E  I  F  M  K  D  L  G  W  D  D  W   120
GTGGTCTATCCCCCGAGCCTCACTTTTGTCCAGTGTGCATTGTGCAACTATGAAACAAACACTGTGCAGTGTCCATCCTCTCACGCCCGCATCCAGGATGACAGCTCACAGGTGCCATGT     619
 V  V  Y  P  P  S  L  T  F  V  Q  C  A  L  C  N  Y  E  T  N  T  V  Q  C  P  S  S  H  A  R  I  Q  D  D  S  S  Q  V  P  C   160
TGTCTGCCCCACTCCAAGGAAATGGTGCCAGTCGTCTACGTGGATGAAACTGGCACTGTGGTCCTTTCCTCCGTGTATCTGACCAGGAGCTGCAGCTGTGAGGCTGCAAACATCCAGCAG     739
 C  L  P  H  S  K  E  M  V  P  V  V  Y  V  D  E  T  G  T  V  V  L  S  S  V  Y  L  T  R  S  C  S  C  E  A  A  N  I  Q  Q   200
CCAGGCACAGAGTAAagtttgttttgtcagatcatggcaactgtgagccacctggcacaaaaacaaaattcagaaaaagtagataacaacctccaaatgcagactaatcttctcctacga     859
 P  G  T  E  *                                                                                                            204
tgcatgttgctttttccatgttcactgtgggaaactaaagcagggctatttcagtttggcattcctgtttcccgtgttacgaccagagagaatgatacagtgttgaaatgtatttgtaaa     979
atcatgttatatgatttgctgattttatgttgtagcttgtttacctccaagcttatgaatttagagaactatgattacggccggacttggttacttggtgttccattcaggaatctgaca     1099
ctggttggagccaaatactctgatttgccagttgtctcctttctgcactgtgagtcaattccatcccaccatggaaaataaactgcaaatcactggcaaaaaaaaaaaaaaaaaaaaaaa     1219
aaaaa                                                                                                                      1224
```

Supplementary Figure 1. Full-length cDNA sequence and the deduced amino acids of *CS-Gsdf*. The deduced amino acid residues are represented as single-letter abbreviations. Open reading frame and untranslated regions are indicated in uppercase and lowercase, respectively. The stop codon (TAA) is indicated by an asterisk. The polyadenylation signal and Poly-A tail are marked by a box and underline, respectively.

MSFAFSAMLMLLGSSVVLAFVXQPSHEKAAVSSMSRNRCQFESVDIKKHLL
RELNLQTEPRLPAGGLDALRERWQRNFGALSDRTTDISVATTRRGDRRNLEC
CAMTSEIFMKDLGWDDWVVYPPSLTFVQCALCNYETNTVQCPSSHARIQD
DSSQVPCCLPHSKEMVPVVYVDETGTVVLSSVYLTRSCSCEAANIQQPGTE

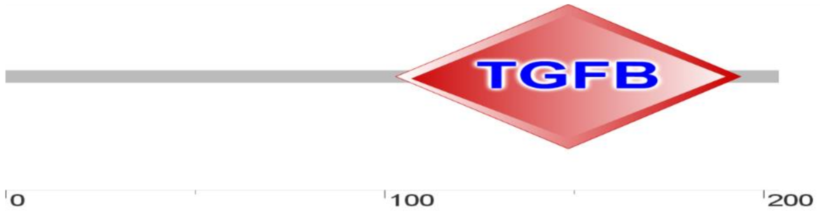

Supplementary Figure 2. Deduced protein sequence and conserved domain of *CS-Gsdf*. The predicted TGF-β domain is a multifunctional peptide that controls proliferation, differentiation, and other functions in many cell types. Transforming growth factor-beta (TGF-beta) family glycoprotein hormones and platelet-derived growth factor.

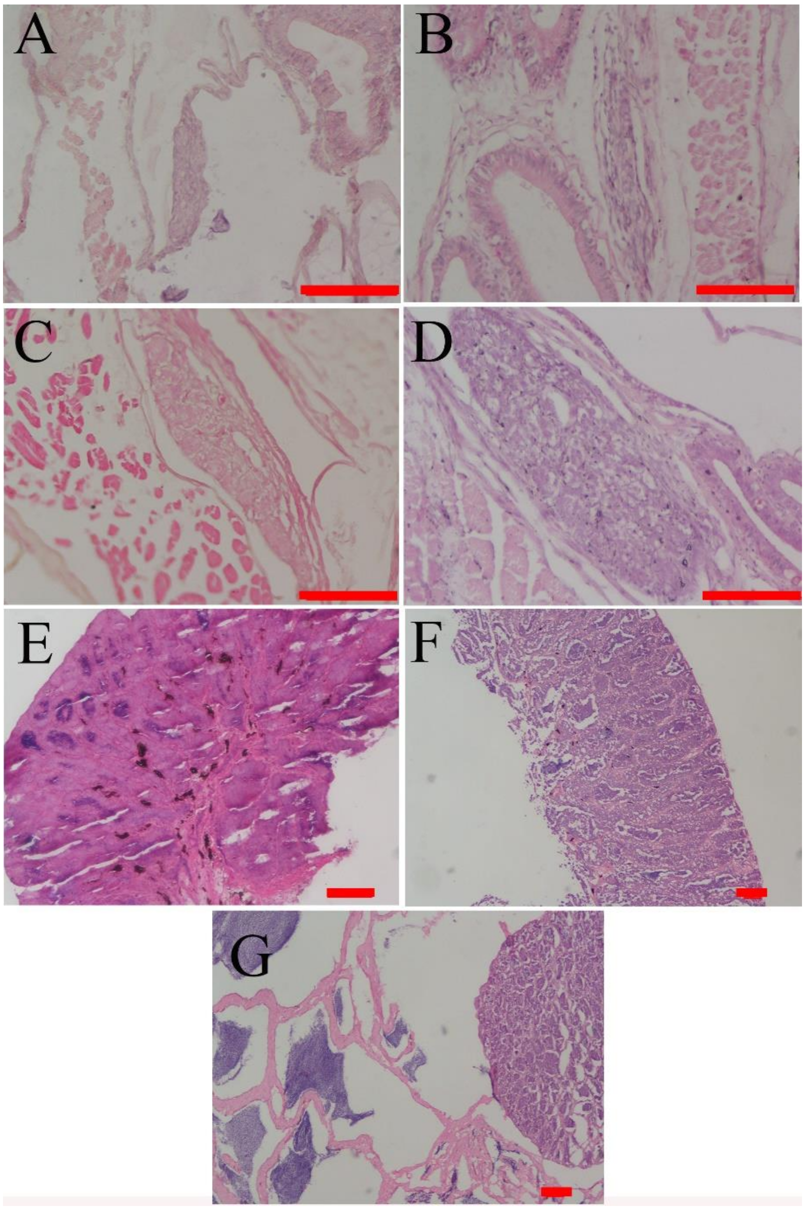

Supplementary Figure 3. The histologic analysis about the different stages of male gonad development. Panels A, B, C, and D show the 20 dph, 35 dph, 65 dph and 86 dph gonads of male, respectively; E, F and G show the 120dph, 1yph and 2yph testes, respectively. Scale bars: 100 μm.

Supplementary Table 1. Primers used in this study

**Primer**	**Sequences (5'-3')**	**Primer usage**
Gsdf(ish) **F**	GAAGAATTCTCTTCGGTTGTGCTTGCG	ISH
Gsdf(ish) **R**	GACAAGCTTATTTCCTTGGAGTGGGGC	ISH
Gsdf(dl) **F**	TGTGCTTGCGTTTGTCTTGC	qRT-PCR
Gsdf(dl) **R**	CACTCCAGGTTCCTTCTGTCTCC	qRT-PCR
β-actin **F**	CCTTGGTATGGAGTCCTGTGGC	qRT-PCR
β-actin **R**	TCCTTCTGCATCCTGTCGGTC	qRT-PCR
Star **F**	AGGACGGCTGGACCACTGAAAT	qRT-PCR
Star **R**	ACCTCGTGGGTGACCATCGTGT	qRT-PCR
Foxl2 **F**	GGATACGGGCGTGGTGAA	qRT-PCR
Foxl2 **R**	TGAATGGCCGGGTGCTTA	qRT-PCR
Wnt4a **F**	TAAAGGTCAGTCTTCCAGCCGA	qRT-PCR
Wnt4a **R**	TGGCACCATCAAACTTCTCCTT	qRT-PCR
Cyp19a **F**	ATACGGGCGTGGTGAATTG	qRT-PCR
Cyp19a **R**	TGAATGGCCGGGTGCGGC	qRT-PCR
scaffold68-2 **F**	ATTCACTGACCCCTGAGAGC	Genetic sex determination
scaffold68-2 **R**	TGGCACCATCATTGTAAAACTA	Genetic sex determination
